# Ammonium 2-(3,4-di­methyl­benzo­yl)benzoate dihydrate

**DOI:** 10.1107/S1600536813012087

**Published:** 2013-05-11

**Authors:** Ming-Hui Zhang, Yue-Lin Yuan, Jun-Feng Kou

**Affiliations:** a College of Chemistry and Chemical Engineering, Yunnan Normal University, Kunming 650500, People’s Republic of China

## Abstract

In the anion of the title compound, NH_4_^+^·C_16_H_13_O_3_^−^·2H_2_O, the benzene rings are twisted with respect to each other by 73.56 (10)°. In the crystal, extensive N—H⋯O and O—H⋯O hydrogen bonds link the cations, anions and lattice water mol­ecules into a three dimensional supra­molecular structure.

## Related literature

For the synthesis of the title compound, see: Elofson *et al.* (1965 ▶). For related compounds, see: Boon *et al.* (1986 ▶); Yeung *et al.* (2002 ▶); Gopalakrishnan *et al.* (2005 ▶); Qiao *et al.* (2008 ▶); Gouda *et al.* (2010 ▶).
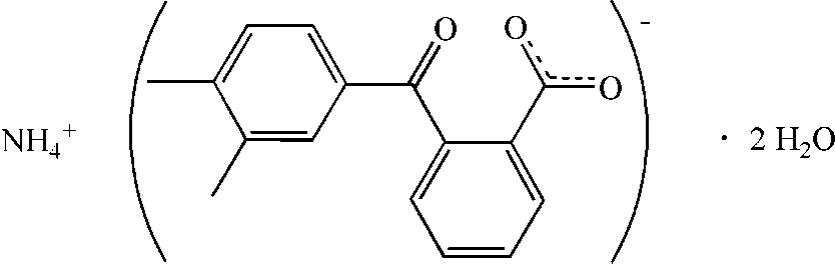


## Experimental

### 

#### Crystal data

NH_4_^+^·C_16_H_13_O_3_^−^·2H_2_O
*M* *_r_* = 307.34
Triclinic, 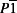

*a* = 7.5039 (15) Å
*b* = 7.7458 (15) Å
*c* = 14.439 (3) Å
α = 81.63 (3)°
β = 79.15 (3)°
γ = 78.67 (3)°
*V* = 803.0 (3) Å^3^
*Z* = 2
Mo *K*α radiation
μ = 0.09 mm^−1^
*T* = 293 K
0.20 × 0.18 × 0.15 mm

#### Data collection

Rigaku MM007-HF CCD (Saturn 724+) diffractometer
6289 measured reflections
2791 independent reflections
1674 reflections with *I* > 2σ(*I*)
*R*_int_ = 0.032

#### Refinement

*R*[*F* ^2^ > 2σ(*F* ^2^)] = 0.040
*wR*(*F* ^2^) = 0.114
*S* = 1.01
2791 reflections
201 parameters
H-atom parameters constrained
Δρ_max_ = 0.33 e Å^−3^
Δρ_min_ = −0.19 e Å^−3^

### 

Data collection: *CrystalStructure* (Rigaku/MSC, 2006 ▶); cell refinement: *CrystalStructure*; data reduction: *CrystalStructure*; program(s) used to solve structure: *SHELXTL* (Sheldrick, 2008 ▶); program(s) used to refine structure: *SHELXTL*; molecular graphics: *DIAMOND* (Brandenburg, 1999 ▶); software used to prepare material for publication: *SHELXTL*.

## Supplementary Material

Crystal structure: contains datablock(s) I, new_global_publ_block. DOI: 10.1107/S1600536813012087/xu5697sup1.cif

Structure factors: contains datablock(s) I. DOI: 10.1107/S1600536813012087/xu5697Isup2.hkl

Additional supplementary materials:  crystallographic information; 3D view; checkCIF report

## Figures and Tables

**Table 1:** Hydrogen-bond geometry (Å, °)

*D*—H⋯*A*	*D*—H	H⋯*A*	*D*⋯*A*	*D*—H⋯*A*
N1—H1*A*⋯O4*W*	0.93	1.98	2.836 (2)	152
N1—H1*B*⋯O3	0.93	1.90	2.820 (2)	170
N1—H1*C*⋯O3^i^	0.96	1.88	2.823 (3)	167
N1—H1*D*⋯O5*W* ^ii^	0.96	2.03	2.871 (3)	144
N1—H1*D*⋯O4*W* ^iii^	0.96	2.45	3.067 (3)	121
O4*W*—H4*WA*⋯O2^iv^	0.90	1.93	2.809 (2)	164
O4*W*—H4*WB*⋯O2^ii^	0.90	1.91	2.808 (2)	172
O5*W*—H5*WA*⋯O1^v^	0.87	2.04	2.899 (2)	171
O5*W*—H5*WB*⋯O2	0.85	2.31	3.032 (2)	142

Symmetry codes: (i) 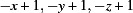; (ii) 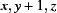; (iii) 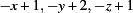; (iv) 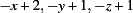; (v) 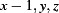.

**Fig. 1.**
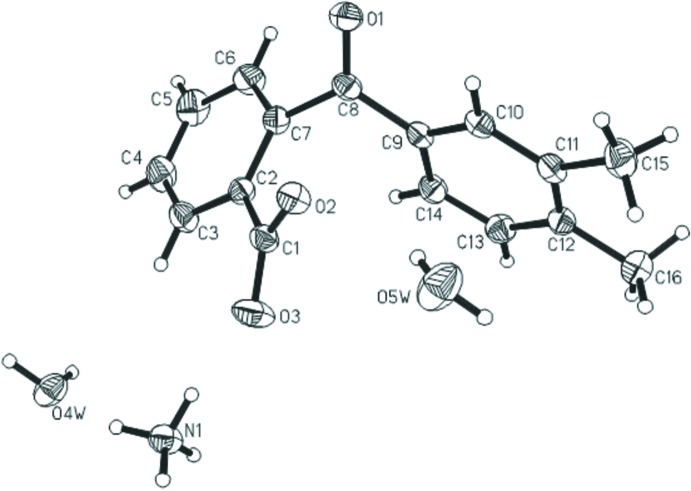
The molecular structure of the title compound, with atom labels and 30% probability displacement ellipsoids.

**Fig. 2.**
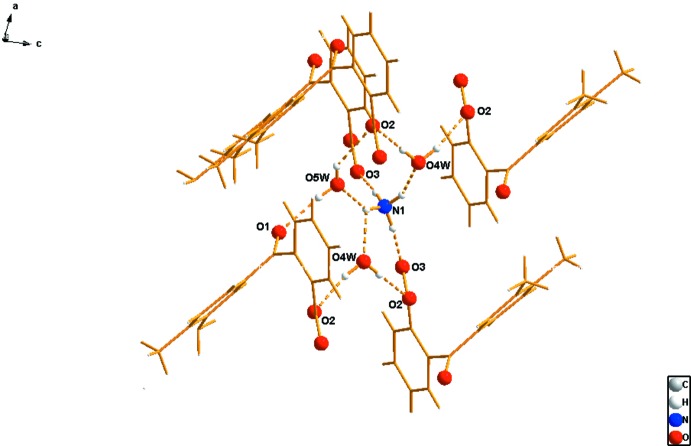
Hydrogen bonds are shown as brown dashed lines.

**Fig. 3.**
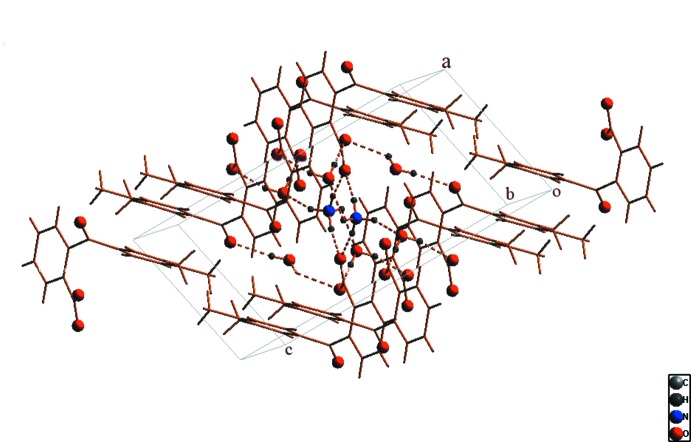
A view of the crystal packing.

## supplementary crystallographic information

### Comment

Friedel-Crafts acylation provides a fundamentaland useful method for the synthesis of aromatic ketones, which are important intermediates for preparing fine chemicals in the field of pharmaceuticals, agrochemicals, and fragrances. Typically, these reactions are performed using acyl chloride (for acylation) in the presence of a little more than one equivalent of Lewis acids, such as anhydrous aluminium chloride, titanium chloride and iron chloride (Elofson *et al.* 1965; Boon *et al.* 1986; Yeung *et al.* 2002; Gopalakrishnan *et al.* 2005; Qiao *et al.* 2008; Gouda *et al.* 2010). Herein we report the synthesis and structure of the title compound with aluminium chloride.

The structure of the title compound is shown in Fig. 1, Fig. 2 and hydrogen-bond geometry is given in Table 1. The compound crystallizes in the triclinic space group *p*-1,and the crystallographic asymmetric unit consists of one crystallo graphically independent anion, one ammonium cation and two water molecules. As shown in Fig.2, the dihedral angle is 73.2 between benzene rings which are not coplane. An interesting part of the structure of title compound is the helical chains formed by the N—H···O and O—H···O. hydrogen-bonding interactions along *c* axis in this molecule (Table 1 & Fig.3). Two neighouring N atoms formed helical chain (O3—N1—O4W—O2 andO2-O4W—N1—O3) which bridge two carboxy O3 atoms formed two oppposite handed chains with the bond distances of N1···O3 2.823 (3) Å, N1···O4w 3.067 (3) Å, O4W···O2 2.808 (2) Å. Further connection of the helices *via* N—H···O5W hydrogen bond with the bond distance of 2.871 Å gives the three-dimensional structure.

### Experimental

In a 250 ml dry three-necked round-bottom flask, aluminium chloride (34.8 g, 0.26 mol) was dissolved in dry dichloromethane (150 ml), *ortho*-xylene (11.2 g, 0.105 mol) was added and then phthalic anhydride portion-wise with formation of an orange liquid in ice bath for 3 h while stirring. The mixture was reacted for 10 h while elevating the temperature of the reactor up to 303 K with formation of a yellow precipitate, then cooled down to room temperature and poured over a mixture of ice (20 g) and concentrated hydrochloric acid (10 ml) with a large amount of gas generated by HCl (Elofson *et al.*, 1965). The organic layer was separated and the aqueous layer was extracted with dichloromethane (3 × 50 ml). The combined organic layers were washed with water (20 ml), concentrated in the rotary evaporator to give the pure yell compound, dried in vaccum and yield: 20.2 g, 80%. MS(EI): m/*z* = 254([M—CH3]+). Crystals suitable for X-ray analysis were obtained by evaporate slowly the solution of the compound in 25% aqueous ammonia in 88% yield within a month.

### Refinement

H atoms attached to carbons were geometrically fixed and allowed to ride on the corresponding non-H atom with C—H = 0.96 Å, and *U*_iso_(H) = 1.5*U*_eq_(C) of the attached C atom for methyl H atoms and 1.2*U*_eq_(C) for other H atoms. The H atoms were constrained with N—H distances of 0.93–0.96 Å, *U*_iso_(H) = 1.2*U*_eq_(N) and O—H distance of 0.85–0.90 Å with *U*_iso_(H) = 1.2*U*_eq_(O).

### Crystal data

**Table:**

NH_4_^+^·C_16_H_13_O_3_^−^·2H_2_O	*Z* = 2
*M**_r_* = 307.34	*F*(000) = 328
Triclinic, *P*1	*D*_x_ = 1.271 Mg m^−^^3^
Hall symbol: -P 1	Mo *K*α radiation, λ = 0.71073 Å
*a* = 7.5039 (15) Å	Cell parameters from 9573 reflections
*b* = 7.7458 (15) Å	θ = 3.2–25.0°
*c* = 14.439 (3) Å	µ = 0.09 mm^−^^1^
α = 81.63 (3)°	*T* = 293 K
β = 79.15 (3)°	Block, colorless
γ = 78.67 (3)°	0.20 × 0.18 × 0.15 mm
*V* = 803.0 (3) Å^3^	

### Data collection

**Table:**

Rigaku MM007-HF CCD (Saturn 724+) diffractometer	1674 reflections with *I* > 2σ(*I*)
Radiation source: rotating anode	*R*_int_ = 0.032
Confocal monochromator	θ_max_ = 25.0°, θ_min_ = 3.2°
ω scans at fixed χ = 45°	*h* = −8→8
6289 measured reflections	*k* = −9→8
2791 independent reflections	*l* = −17→16

### Refinement

**Table:**

Refinement on *F*^2^	Primary atom site location: structure-invariant direct methods
Least-squares matrix: full	Secondary atom site location: difference Fourier map
*R*[*F*^2^ > 2σ(*F*^2^)] = 0.040	Hydrogen site location: inferred from neighbouring sites
*wR*(*F*^2^) = 0.114	H-atom parameters constrained
*S* = 1.01	*w* = 1/[σ^2^(*F*_o_^2^) + (0.0585*P*)^2^ + 0.0127*P*] where *P* = (*F*_o_^2^ + 2*F*_c_^2^)/3
2791 reflections	(Δ/σ)_max_ < 0.001
201 parameters	Δρ_max_ = 0.33 e Å^−^^3^
0 restraints	Δρ_min_ = −0.19 e Å^−^^3^

### Special details

**Table:**

Geometry. All e.s.d.'s (except the e.s.d. in the dihedral angle between two l.s. planes) are estimated using the full covariance matrix. The cell e.s.d.'s are taken into account individually in the estimation of e.s.d.'s in distances, angles and torsion angles; correlations between e.s.d.'s in cell parameters are only used when they are defined by crystal symmetry. An approximate (isotropic) treatment of cell e.s.d.'s is used for estimating e.s.d.'s involving l.s. planes.
Refinement. Refinement of *F*^2^ against ALL reflections. The weighted *R*-factor *wR* and goodness of fit *S* are based on *F*^2^, conventional *R*-factors *R* are based on *F*, with *F* set to zero for negative *F*^2^. The threshold expression of *F*^2^ > σ(*F*^2^) is used only for calculating *R*-factors(gt) *etc*. and is not relevant to the choice of reflections for refinement. *R*-factors based on *F*^2^ are statistically about twice as large as those based on *F*, and *R*- factors based on ALL data will be even larger.

### Fractional atomic coordinates and isotropic or equivalent isotropic displacement parameters (Å^2^)

**Table:**

	*x*	*y*	*z*	*U*_iso_*/*U*_eq_	
C1	0.8894 (3)	0.3453 (3)	0.37419 (13)	0.0384 (5)	
C2	1.0501 (3)	0.4382 (2)	0.33412 (13)	0.0358 (5)	
C3	1.0619 (3)	0.5954 (3)	0.36741 (15)	0.0481 (6)	
H3	0.9645	0.6478	0.4100	0.058*	
C4	1.2161 (3)	0.6742 (3)	0.33795 (17)	0.0568 (6)	
H4	1.2218	0.7789	0.3608	0.068*	
C5	1.3603 (3)	0.5986 (3)	0.27530 (17)	0.0596 (7)	
H5	1.4644	0.6514	0.2560	0.071*	
C6	1.3514 (3)	0.4435 (3)	0.24053 (16)	0.0502 (6)	
H6	1.4501	0.3921	0.1982	0.060*	
C7	1.1961 (3)	0.3644 (2)	0.26844 (13)	0.0370 (5)	
C8	1.1918 (3)	0.2019 (3)	0.22316 (13)	0.0379 (5)	
C9	1.0404 (3)	0.2066 (2)	0.16934 (12)	0.0350 (5)	
C10	0.9867 (3)	0.0489 (3)	0.15866 (13)	0.0386 (5)	
H10	1.0460	−0.0577	0.1862	0.046*	
C11	0.8476 (3)	0.0471 (3)	0.10814 (13)	0.0400 (5)	
C12	0.7643 (3)	0.2059 (3)	0.06282 (14)	0.0415 (5)	
C13	0.8184 (3)	0.3637 (3)	0.07373 (14)	0.0458 (5)	
H13	0.7633	0.4702	0.0441	0.055*	
C14	0.9519 (3)	0.3644 (3)	0.12749 (14)	0.0416 (5)	
H14	0.9825	0.4712	0.1357	0.050*	
C15	0.7866 (3)	−0.1259 (3)	0.10343 (17)	0.0578 (6)	
H15A	0.8145	−0.1540	0.0391	0.087*	
H15B	0.6563	−0.1148	0.1251	0.087*	
H15C	0.8504	−0.2188	0.1431	0.087*	
C16	0.6165 (3)	0.2111 (3)	0.00431 (17)	0.0592 (6)	
H16A	0.5107	0.1731	0.0444	0.089*	
H16B	0.6623	0.1335	−0.0442	0.089*	
H16C	0.5823	0.3298	−0.0245	0.089*	
N1	0.5486 (2)	0.7384 (2)	0.49120 (13)	0.0498 (5)	
H1A	0.6138	0.7907	0.5245	0.060*	
H1B	0.6234	0.6452	0.4616	0.060*	
H1C	0.4464	0.6937	0.5318	0.060*	
H1D	0.5135	0.8306	0.4423	0.060*	
O1	1.3187 (2)	0.07641 (19)	0.22420 (10)	0.0517 (4)	
O2	0.91984 (18)	0.17891 (17)	0.37486 (9)	0.0428 (4)	
O3	0.7384 (2)	0.4340 (2)	0.40630 (12)	0.0634 (5)	
O4W	0.7770 (2)	0.9592 (2)	0.53226 (11)	0.0572 (4)	
H4WA	0.8648	0.9304	0.5697	0.069*	
H4WB	0.8282	1.0201	0.4789	0.069*	
O5W	0.6084 (2)	0.0140 (3)	0.33873 (12)	0.0833 (6)	
H5WA	0.5287	0.0395	0.3001	0.100*	
H5WB	0.6978	0.0708	0.3198	0.100*	

### Atomic displacement parameters (Å^2^)

**Table:**

	*U*^11^	*U*^22^	*U*^33^	*U*^12^	*U*^13^	*U*^23^
C1	0.0357 (12)	0.0423 (13)	0.0370 (11)	−0.0088 (10)	0.0010 (9)	−0.0099 (9)
C2	0.0341 (11)	0.0372 (11)	0.0374 (10)	−0.0096 (9)	−0.0047 (9)	−0.0054 (9)
C3	0.0511 (14)	0.0460 (13)	0.0494 (13)	−0.0131 (11)	−0.0024 (10)	−0.0137 (10)
C4	0.0656 (17)	0.0513 (14)	0.0617 (15)	−0.0262 (12)	−0.0087 (13)	−0.0142 (12)
C5	0.0577 (16)	0.0622 (17)	0.0661 (15)	−0.0360 (13)	−0.0048 (13)	−0.0033 (12)
C6	0.0406 (13)	0.0568 (14)	0.0533 (13)	−0.0181 (11)	0.0046 (10)	−0.0098 (11)
C7	0.0356 (12)	0.0405 (12)	0.0361 (11)	−0.0122 (9)	−0.0038 (9)	−0.0031 (9)
C8	0.0357 (12)	0.0396 (12)	0.0362 (11)	−0.0091 (10)	0.0038 (9)	−0.0056 (9)
C9	0.0370 (11)	0.0337 (11)	0.0331 (10)	−0.0060 (9)	0.0011 (9)	−0.0089 (9)
C10	0.0433 (12)	0.0311 (11)	0.0389 (11)	−0.0037 (9)	−0.0021 (9)	−0.0056 (9)
C11	0.0442 (12)	0.0364 (12)	0.0392 (11)	−0.0092 (10)	0.0004 (10)	−0.0099 (9)
C12	0.0372 (12)	0.0505 (13)	0.0372 (11)	−0.0090 (10)	−0.0008 (9)	−0.0112 (10)
C13	0.0486 (14)	0.0404 (12)	0.0463 (12)	−0.0035 (10)	−0.0089 (10)	−0.0027 (10)
C14	0.0475 (13)	0.0340 (12)	0.0440 (12)	−0.0088 (10)	−0.0049 (10)	−0.0073 (9)
C15	0.0688 (17)	0.0475 (14)	0.0633 (15)	−0.0197 (12)	−0.0099 (12)	−0.0143 (11)
C16	0.0538 (15)	0.0681 (17)	0.0600 (14)	−0.0092 (12)	−0.0171 (12)	−0.0133 (13)
N1	0.0438 (11)	0.0422 (10)	0.0637 (12)	−0.0110 (8)	0.0008 (9)	−0.0148 (9)
O1	0.0425 (9)	0.0480 (9)	0.0634 (10)	0.0012 (7)	−0.0080 (7)	−0.0155 (8)
O2	0.0434 (9)	0.0356 (8)	0.0480 (8)	−0.0117 (6)	0.0002 (7)	−0.0039 (6)
O3	0.0411 (10)	0.0552 (10)	0.0904 (12)	−0.0113 (8)	0.0169 (8)	−0.0289 (9)
O4W	0.0463 (9)	0.0669 (10)	0.0606 (9)	−0.0221 (8)	−0.0130 (7)	0.0081 (8)
O5W	0.0654 (12)	0.1233 (16)	0.0654 (11)	−0.0396 (12)	−0.0147 (9)	0.0123 (11)

### Geometric parameters (Å, º)

**Table:**

C1—O3	1.249 (2)	C11—C15	1.513 (3)
C1—O2	1.263 (2)	C12—C13	1.399 (3)
C1—C2	1.505 (3)	C12—C16	1.507 (3)
C2—C3	1.396 (2)	C13—C14	1.379 (3)
C2—C7	1.395 (3)	C13—H13	0.9300
C3—C4	1.382 (3)	C14—H14	0.9300
C3—H3	0.9300	C15—H15A	0.9600
C4—C5	1.367 (3)	C15—H15B	0.9600
C4—H4	0.9300	C15—H15C	0.9600
C5—C6	1.386 (3)	C16—H16A	0.9600
C5—H5	0.9300	C16—H16B	0.9600
C6—C7	1.389 (3)	C16—H16C	0.9600
C6—H6	0.9300	N1—H1A	0.9264
C7—C8	1.509 (3)	N1—H1B	0.9263
C8—O1	1.220 (2)	N1—H1C	0.9630
C8—C9	1.484 (3)	N1—H1D	0.9636
C9—C14	1.385 (3)	O4W—H4WA	0.9042
C9—C10	1.396 (3)	O4W—H4WB	0.9039
C10—C11	1.385 (3)	O5W—H5WA	0.8692
C10—H10	0.9300	O5W—H5WB	0.8542
C11—C12	1.396 (3)		
			
O3—C1—O2	124.36 (19)	C10—C11—C15	119.80 (19)
O3—C1—C2	119.25 (18)	C12—C11—C15	120.91 (18)
O2—C1—C2	116.36 (17)	C11—C12—C13	118.71 (17)
C3—C2—C7	118.41 (18)	C11—C12—C16	121.57 (18)
C3—C2—C1	120.09 (18)	C13—C12—C16	119.7 (2)
C7—C2—C1	121.35 (16)	C14—C13—C12	121.3 (2)
C4—C3—C2	121.0 (2)	C14—C13—H13	119.3
C4—C3—H3	119.5	C12—C13—H13	119.3
C2—C3—H3	119.5	C13—C14—C9	120.21 (17)
C5—C4—C3	120.15 (19)	C13—C14—H14	119.9
C5—C4—H4	119.9	C9—C14—H14	119.9
C3—C4—H4	119.9	C11—C15—H15A	109.5
C4—C5—C6	120.0 (2)	C11—C15—H15B	109.5
C4—C5—H5	120.0	H15A—C15—H15B	109.5
C6—C5—H5	120.0	C11—C15—H15C	109.5
C5—C6—C7	120.4 (2)	H15A—C15—H15C	109.5
C5—C6—H6	119.8	H15B—C15—H15C	109.5
C7—C6—H6	119.8	C12—C16—H16A	109.5
C6—C7—C2	119.96 (17)	C12—C16—H16B	109.5
C6—C7—C8	117.37 (18)	H16A—C16—H16B	109.5
C2—C7—C8	122.65 (17)	C12—C16—H16C	109.5
O1—C8—C9	121.17 (16)	H16A—C16—H16C	109.5
O1—C8—C7	120.19 (17)	H16B—C16—H16C	109.5
C9—C8—C7	118.34 (17)	H1A—N1—H1B	111.3
C14—C9—C10	118.58 (17)	H1A—N1—H1C	111.9
C14—C9—C8	121.56 (16)	H1B—N1—H1C	108.1
C10—C9—C8	119.85 (18)	H1A—N1—H1D	103.8
C11—C10—C9	121.76 (19)	H1B—N1—H1D	107.6
C11—C10—H10	119.1	H1C—N1—H1D	114.1
C9—C10—H10	119.1	H4WA—O4W—H4WB	105.6
C10—C11—C12	119.28 (17)	H5WA—O5W—H5WB	111.4
			
O3—C1—C2—C3	28.0 (3)	C2—C7—C8—C9	57.7 (3)
O2—C1—C2—C3	−150.26 (18)	O1—C8—C9—C14	−147.4 (2)
O3—C1—C2—C7	−156.54 (19)	C7—C8—C9—C14	26.3 (3)
O2—C1—C2—C7	25.2 (3)	O1—C8—C9—C10	31.4 (3)
C7—C2—C3—C4	−1.5 (3)	C7—C8—C9—C10	−154.87 (18)
C1—C2—C3—C4	174.1 (2)	C14—C9—C10—C11	−0.4 (3)
C2—C3—C4—C5	0.0 (3)	C8—C9—C10—C11	−179.27 (17)
C3—C4—C5—C6	0.6 (4)	C9—C10—C11—C12	3.1 (3)
C4—C5—C6—C7	0.4 (3)	C9—C10—C11—C15	−176.39 (18)
C5—C6—C7—C2	−2.0 (3)	C10—C11—C12—C13	−3.0 (3)
C5—C6—C7—C8	176.4 (2)	C15—C11—C12—C13	176.5 (2)
C3—C2—C7—C6	2.5 (3)	C10—C11—C12—C16	178.04 (19)
C1—C2—C7—C6	−173.04 (19)	C15—C11—C12—C16	−2.5 (3)
C3—C2—C7—C8	−175.84 (18)	C11—C12—C13—C14	0.4 (3)
C1—C2—C7—C8	8.6 (3)	C16—C12—C13—C14	179.34 (19)
C6—C7—C8—O1	53.1 (3)	C12—C13—C14—C9	2.3 (3)
C2—C7—C8—O1	−128.6 (2)	C10—C9—C14—C13	−2.3 (3)
C6—C7—C8—C9	−120.7 (2)	C8—C9—C14—C13	176.55 (18)

### Hydrogen-bond geometry (Å, º)

**Table:**

*D*—H···*A*	*D*—H	H···*A*	*D*···*A*	*D*—H···*A*
N1—H1*A*···O4*W*	0.93	1.98	2.836 (2)	152
N1—H1*B*···O3	0.93	1.90	2.820 (2)	170
N1—H1*C*···O3^i^	0.96	1.88	2.823 (3)	167
N1—H1*D*···O5*W*^ii^	0.96	2.03	2.871 (3)	144
N1—H1*D*···O4*W*^iii^	0.96	2.45	3.067 (3)	121
O4*W*—H4*WA*···O2^iv^	0.90	1.93	2.809 (2)	164
O4*W*—H4*WB*···O2^ii^	0.90	1.91	2.808 (2)	172
O5*W*—H5*WA*···O1^v^	0.87	2.04	2.899 (2)	171
O5*W*—H5*WB*···O2	0.85	2.31	3.032 (2)	142

Symmetry codes: (i) −*x*+1, −*y*+1, −*z*+1; (ii) *x*, *y*+1, *z*; (iii) −*x*+1, −*y*+2, −*z*+1; (iv) −*x*+2, −*y*+1, −*z*+1; (v) *x*−1, *y*, *z*.
